# Enhanced Methane Emissions during Amazonian Drought by Biomass Burning

**DOI:** 10.1371/journal.pone.0166039

**Published:** 2016-11-16

**Authors:** Makoto Saito, Heon-Sook Kim, Akihiko Ito, Tatsuya Yokota, Shamil Maksyutov

**Affiliations:** Center for Global Environmental Research, National Institute for Environmental Studies, 16-2 Onogawa, Tsukuba, Ibaraki 305-8506, Japan; CAS, CHINA

## Abstract

The Amazon is a significant source of atmospheric methane, but little is known about the source response to increasing drought severity and frequency. We investigated satellite observations of atmospheric column-averaged methane for the 2010 drought and subsequent 2011 wet year in the Amazon using an atmospheric inversion scheme. Our analysis indicates an increase in atmospheric methane over the southern Amazon region during the drought, representing an increase in annual emissions relative to the wet year. We attribute the increase to emissions from biomass burning driven by intense drought, combined with carbon monoxide showing seasonal variations corresponding to methane variations. We show that there is probably a strong correspondence between drought and methane emissions in the Amazon.

## Introduction

The Amazon region plays an important role in the global carbon cycle as a large and dynamic reservoir of organic carbon stored in forest biomass and soil [1], and as a significant source of atmospheric methane (CH_4_), accounting for ∼10% of atmospheric contributions globally [2]. The Amazon region suffered severe droughts in 2005 and 2010, caused by factors related to higher than average sea surface temperatures in the tropical North Atlantic, weaker trade winds, lower than average water vapor transport into the southern Amazon region, and a weakening of upward atmospheric flow over the region [3]. The drought events illustrate the vulnerability of forests to climate variations and the impact of intense moisture deficits on canopy structure, leading to a significant net loss of carbon [4]. The risk and occurrence of high-intensity forest fires is increased during droughts, thus accelerating carbon emissions [5]. Carbon dioxide (CO_2_) emitted to the atmosphere is balanced in part by subsequent regrowth of vegetation, whereas CH_4_ release is not. Climate models predict that the intensity and frequency of Amazonian droughts will increase rapidly in the 21st century [6]. Therefore, analyses of the 2005 and 2010 droughts could provide a better understanding of the role of Amazonian forests in the global carbon cycle under near-future climate conditions.

Amazonian wetlands are a primary source of atmospheric CH_4_ [7]. The Amazonian CH_4_ budget includes multiple sources and sinks (i.e., rivers and upland soils) whose contributions are sensitive to feedback from drought conditions. Field measurements under experimental drought conditions provide estimates of the degree of sensitivity on some sources. However, significant gaps remain in our understanding of how CH_4_ budgets in the Amazon region will respond to drought events, in part because observations representative of the entire region are lacking, which are required to reliably assess regional-scale CH_4_ budgets.

**Fig 1:**
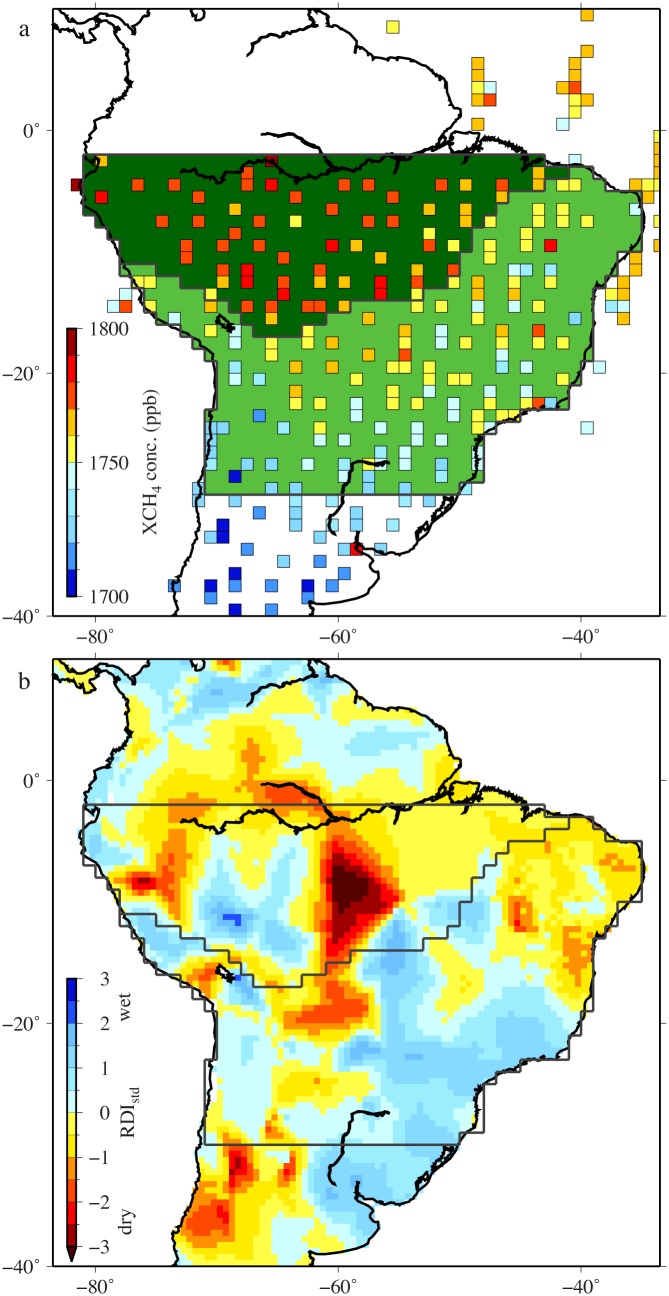
(a) Distribution of averaged concentrations of XCH_4_ (ppb) from observational data acquired by GOSAT for August and September 2010 at a 1° × 1° grid resolution, with 479 and 1582 observed concentrations for the southern Amazon and central South America regions, respectively. The two regions analyzed are shown in dark green (southern Amazon region) and light green (central South America region). (b) Distribution of the standardized Reconnaissance Drought Index (RDI_std_) in October 2009–September 2010 calculated using the method of [9] with monthly precipitation and potential evapotranspiration data from the Climate Research Unit (CRU), University of East Anglia, United Kingdom, using 0.5° × 0.5° mean monthly climatology CRU TS3.22 [10]. Both XCH_4_ concentrations and RDI_std_ values are color coded.

Here, we examine the relative importance of drought on the CH_4_ budget in the Amazon region, with a focus on major emission sources. We used retrieved atmospheric column-averaged CH_4_ (XCH_4_; V02.21) levels from Greenhouse gases Observing SATellite (GOSAT) measurements [8] for 2010 and 2011; in 2010, the Amazon experienced a severe drought, whereas 2011 was relatively wet [5]. The GOSAT measurements provide uniform global coverage and thus show a standardized distribution of CH_4_ concentrations over the Amazon (Fig 1a), allowing an assessment of potential impacts of Amazonian drought on the CH_4_ budget.

## Methods

Using a Bayesian inversion scheme, surface CH_4_ flux estimates for 43 regions globally were optimized by matching XCH_4_ retrievals and surface observations. We excluded the northern Amazon region from the analyses because only a few XCH_4_ retrievals were obtained for this region, owing to the presence of stationary clouds (Fig 1a). However, the signature of the 2010 drought remained strong in the XCH_4_ retrieval data, as the 2010 drought was particularly severe in the southern Amazon region (Fig 1b).

### Overview of GOSAT measurements

The GOSAT has been monitoring atmospheric CO_2_ and CH_4_ concentrations from space since its launch in January 2009 [11]. Measurements are taken by an onboard Thermal And Near-infrared Sensor for carbon Observation (TANSO), consisting of a Fourier Transform Spectrometer (FTS) and a Cloud and Aerosol Imager (CAI) [12]. The TANSO-FTS observes intensities of sunlight reflected or emitted from the Earth’s surface and atmosphere in four spectral bands: 0.758–0.775, 1.56–1.72, 1.92–2.08, and 5.5–14.3 *μ*m. The nadir footprint diameter of the TANSO-FTS is 10.5 km. The TANSO-CAI records an image of the atmosphere and the Earth’s surface in four spectral bands, from the near-ultraviolet to near-infrared: 0.370–0.390, 0.664–0.684, 0.860–0.880, and 1.56–1.65 *μ*m, at spatial resolutions of 0.5, 0.5, 0.5, and 1.5 km, respectively. The TANSO-CAI data are used for cloud screening of the TANSO-FTS data. We used XCH_4_ values from short-wavelength infrared radiance spectra from TANSO-FTS bands 1–3 [8].

### Atmospheric inversions

Surface CH_4_ flux estimates are optimized to detect atmospheric CH_4_ concentrations by minimizing the difference between observed and modeled values, *J*(**s**), using a Bayesian inversion:

$$\begin{array}{c} J\left(\text{s}\right)=\frac{1}{2}\left\{{\left(\text{z}-\text{Hs}\right)}^{\text{T}}{\text{R}}^{-1}(\text{z}-\text{Hs})+{\left(\text{s}-{\text{s}}_{\text{p}}\right)}^{\text{T}}{\text{Q}}^{-1}(\text{s}-{\text{s}}_{\text{p}})\right\} \end{array}$$ (1)

where **z** represents an observation vector; **H** is a matrix of response at each measurement site to emissions from each region; **s** and **s**_**p**_ are vectors of estimated and a priori estimated regional fluxes, respectively; **R** and **Q** are error covariance matrices for the model–data mismatch and a priori flux, respectively; and T is the transpose of a matrix. The elements of **H** were calculated using the atmospheric tracer transport model NIES-TM [13]. The vector of the posterior flux **s**′ and its covariance matrix **Q**′ are given by

$$\begin{array}{c} {\text{s}}^{\prime }={\text{s}}_{\text{p}}+{\left({\text{H}}^{\text{T}}{\text{R}}^{-1}\text{H}+{\text{Q}}^{-1}\right)}^{-1}{\text{H}}^{\text{T}}{\text{R}}^{-1}(\text{z}-\text{Hs}) \end{array}$$ (2)

$$\begin{array}{c} {\text{Q}}^{\prime }={\left({\text{H}}^{\text{T}}{\text{R}}^{-1}\text{H}+{\text{Q}}^{-1}\right)}^{-1} \end{array}$$ (3)

The inverse matrices in Eqs (2) and (3) were solved using LU factorization. We estimated monthly CH_4_ fluxes for 43 regions, including 42 land regions at a sub-continental scale and one global ocean region (Fig A in S1 File). A detailed description of the inversion scheme is given by [14].

Observations of single-shot XCH_4_ retrievals and individual ground-based observations from the World Data Center for Greenhouse Gases (WDCGG) [15] and a monitoring project of greenhouse gases over Siberia [16] were integrated in the inversion (Fig B in S1 File). Biases in XCH_4_ retrievals were corrected to latitudinal and monthly ground-based observations using a second-order polynomial approximation [17] (the mean bias was assumed to be -5.9 ppb, with a standard deviation of 12.6 ppb (V02.XX) [8]). The uncertainty of the XCH_4_ retrievals was set as the difference from the polynomial, with a minimum value of 12 ppb [18]. The uncertainties of the ground-based observation data were given by the mean residual standard deviation (RSD) of the GLOBALVIEW-CH_4_ 2009 dataset [19], with minimum values of the flask sampling and continuous measurement data set as 6 and 20 ppb, respectively.

The inversion scheme consists of the NIES-TM, a module for CH_4_ destruction processes caused by hydroxyl free radicals (OH) [20], and a fixed-lag Kalman smoother with a 4-month lag window [21]. The NIES-TM was implemented using a 2.5° × 2.5° horizontal grid resolution and 32 vertical levels, using a hybrid sigma-isentropic coordinate system. The NIES-TM was driven using 6-hourly climate forcings from the Japan Meteorological Agency Climate Data Assimilation System (JCDAS) [22].

The a priori fluxes were composed of three groups of emission sources: anthropogenic, natural, and biomass burning (Table A in S1 File). The anthropogenic sources were based on the emission inventories of the Emission Database for Global Atmospheric Research (EDGAR) v4.2 [23], and include fuel combustion and fugitive emissions from fuels, industrial processes, and product use, as well as agricultural sources, including animal waste and enteric fermentation, waste treatment, and fossil fuel fires, but excluding rice paddies. Natural sources include emissions from wetlands, rice paddies, and soil sources and sinks, which were based on the Vegetation Integrative SImulator for Trace gases (VISIT) terrestrial biosphere model [24], and also include emissions from termites, which were based on the tracer transport model GISS [25]. Biomass burning was based on the Global Fire Emissions Database (GFED) v3.1 product [26]. The a priori monthly fluxes of these three source groups were prepared at a 1° × 1° spatial resolution, and each group was optimized for the 43 regions in the inversion scheme. Uncertainties in the a priori fluxes were set to 20% of the values for the anthropogenic and biomass burning sources and 50% for the natural sources. After inversion, the optimized emission sources in the four regions (Numbers 9, 10, 15 and 16 in Fig A in S1 File) of South America were aggregated over two large regions: the southern Amazon and central South America.

## Results and Discussion

The dry season in the southern Amazon occurs between July and October. The largest positive temperature and the largest negative precipitation anomalies between 2010 and 2011 occurred in August and September 2010 [5]. The XCH_4_ retrievals for this period show strong latitudinal gradients (Fig 1a) due to differences between CH_4_ emission rates in the Northern and Southern Hemispheres. This agrees with previous measurements from the SCanning Imaging Absorption SpectroMeter for Atmospheric CHartographY (SCIAMACHY) satellite [27]. As confirmed by *in situ* surface measurements [28], longitudinal gradients were also observed in the XCH_4_ retrievals. In the southern Amazon, low-level northeasterly air flows were dominated by year-round trade wind inflows across the northeastern coast of Brazil, flowing from the tropical North Atlantic region [29]. Levels of CH_4_ in the southern Amazon were generally enhanced compared with those in the central South America region, indicating the contributions of upwind emission sources.

**Fig 2:**
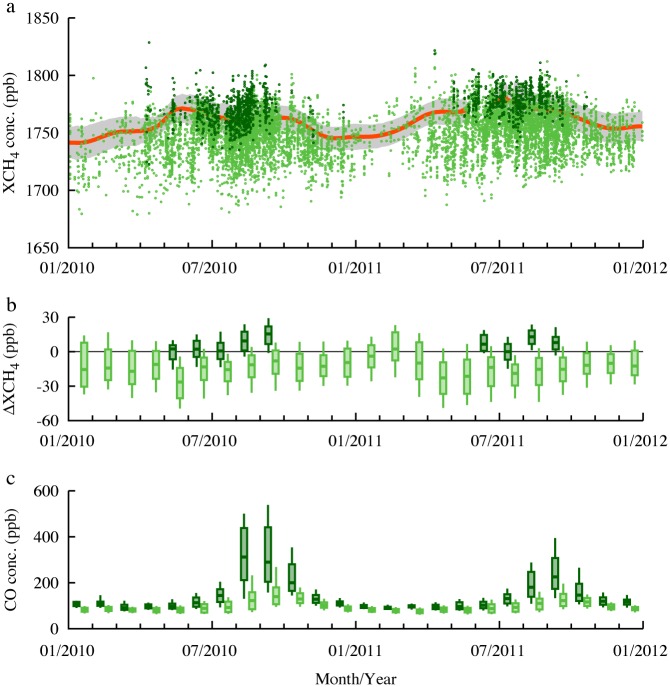
Time series of CH_4_ and CO concentrations for 2010 and 2011. (a) Variability in XCH_4_ concentrations (ppb) in the southern Amazon region (dark green circles), central South America region (light green circles), and background region (ocean area between 0°–20°S and 20°–50°W; red line). Daily data of the background region were constructed from discrete XCH_4_ data using a Humming window with a window-width of 80 days. The standard errors (one sigma) of XCH_4_ data are given by the gray boundary. The discontinuity at the background region for May 22–June 20 in 2011 reflects a lack of XCH_4_ data. (b) Monthly variability in XCH_4_ values (ppb) with the background subtracted (ΔXCH_4_) in the southern Amazon region (dark green box-and-whiskers) and the central South America region (light green box-and-whiskers). Upper and lower values of the boxes are monthly quartiles of ΔXCH_4_, bars through the boxes are medians, and whiskers represent the range of the 10^th^ and 90^th^ percentiles (data are not shown for months in which the number of data points was <25). (c) Same as (b), but showing surface CO concentrations (ppb) from the MOPITT L3 product.

Fig 2 shows the relative effects of drought on atmospheric CH_4_ concentrations, by comparing the variability of XCH_4_ with that of a background region. The Atlantic area between 0–20°S and 20–50°W was determined as the background region for the dominant trade wind. After comparing XCH_4_ with the background observations to obtain the relative effects (ΔXCH_4_), we found a statistically significant difference (*P* < 0.01) between the XCH_4_ variability for July–October in 2010 and 2011 in both the southern Amazon and the central South America regions. Monthly averaged ΔXCH_4_ values increased from July to September 2010 in both regions, with median ΔXCH_4_ values of +14.8 and +7.6 ppb for the southern Amazon and the central South America regions, respectively. In contrast, during 2011 the ΔXCH_4_ levels increased in the southern Amazon region in August and decreased in September. Additionally, in the central South America region, changes in ΔXCH_4_ levels during 2011 were not detectable. The analysis suggests that the 2010 drought enhanced CH_4_ emissions in the southern Amazon region.

**Fig 3:**
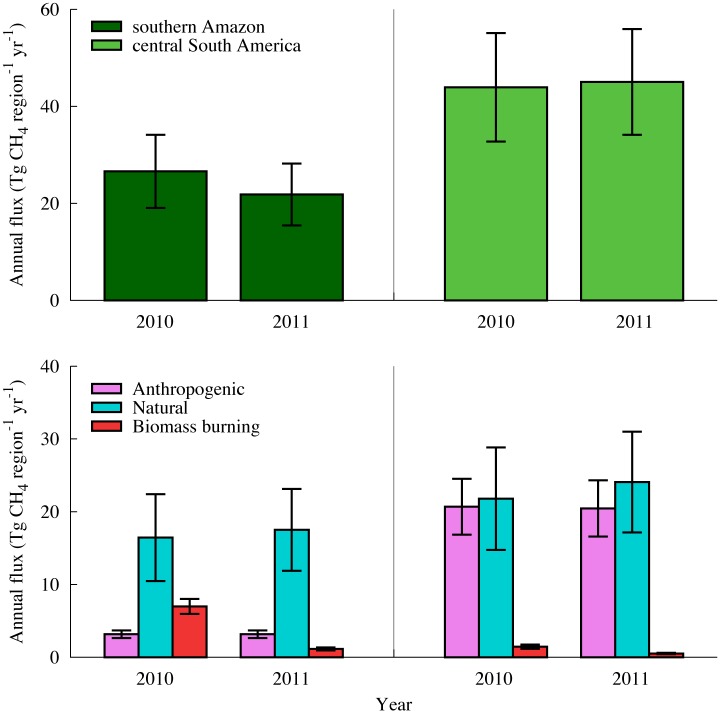
Total annual CH_4_ flux (upper) and relative contributions of anthropogenic (magenta), natural (blue), and biomass-burning (red) fluxes (lower) in the southern Amazon region (left) and central South America region (right) for 2010 and 2011 (in Tg CH_4_ region^−1^ yr^−1^). Error bars represent flux uncertainties.

The inverse model shows that differences in XCH_4_ variability between 2010 and 2011 were closely related to year-to-year changes in emissions from biomass burning (Fig 3). In the southern Amazon region, estimated CH_4_ emissions from biomass burning during 2010 were six times higher than in 2011 (7.0 ± 1.0 versus 1.1 ± 0.2 Tg CH_4_ yr^−1^, respectively, or alternatively given as 1.46 ± 0.21 versus 0.24 ± 0.04 g CH_4_ m^−2^ yr^−1^, respectively). This resulted in a 22% increase in annual CH_4_ fluxes in 2010 relative to those in 2011 (26.6 ± 7.5 versus 21.8 ± 6.4 Tg CH_4_ yr^−1^, respectively) despite a reduction in natural emissions (16.5 ± 6.0 versus 17.5 ± 5.6 Tg CH_4_ yr^−1^, respectively) and no changes in anthropogenic emissions (3.2 ± 0.5 Tg CH_4_ yr^−1^). Enhanced emissions from biomass burning were also detected in the central South America region in 2010 compared with 2011 (1.5 ± 0.3 verses 0.5 ± 0.1 Tg CH_4_ yr^−1^, respectively), but a comparable increase in annual CH_4_ fluxes in 2010 (43.9 ± 11.2 Tg CH_4_ in 2010 verses 45.0 ± 10.9 in 2011 Tg CH_4_) was not discernable because 2011 included increased natural emissions. Annual CH_4_ fluxes over the northern Amazon region in 2010 and 2011 were estimated as 23.9 and 23.0 Tg CH_4_ yr^−1^, respectively, for reference. In 2010, the posterior fluxes led to increases in total emissions of 17% and 9% in the southern Amazon and the central South America regions, respectively, compared with the a priori fluxes (22.6 ± 8.7 and 40.0 ± 13.6 Tg CH_4_ yr^−1^, respectively) (Table A in S1 File).

In the southern Amazon region, the CH_4_ emission rate from biomass burning in 2010 exceeded previous estimates for the Legal Amazon region between 1992 and 1993 of 0.84 g CH_4_ m^−2^ yr^−1^ [30], and for the northern Amazon in 2001–2005 of 0.20 g CH_4_ m^−2^ yr^−1^ (assuming that fire occurs on one third of the days during the 4-month-long dry season) [28], and are comparable to local-scale emissions for primary Amazonian forest between 1986 and 1992 (∼2 g CH_4_ m^−2^ yr^−1^) [31]. Recently [32] estimated the biomass burning emission in the southern Amazon region during 2010 to be 6.8 Tg CH_4_ yr^−1^, using a CH_4_ assimilation system CarbonTracker-CH_4_. Additionally, [33] reported the biomass burning emission of the Amazon Basin during 2010 and 2011 to be 5.4 and 0.5 Tg CH_4_ yr^−1^, respectively, based on the GFED v3.1 product.

The probability distribution for XCH_4_ estimated with these posterior fluxes (PST) for 2010–2011 was compared with XCH_4_ retrievals using the *χ*^2^ goodness-of-fit test, as well as those estimated with a priori fluxes (APR) and posterior fluxes for inversion using ground-based observations only (GB) (Fig C in S1 File). The test for XCH_4_ was divided into 14 classes and yields *χ*^2^ = 151,643 for XCH_4__APR, *χ*^2^ = 1051 for XCH_4__GB, and *χ*^2^ = 451 for XCH_4__PST. The estimated uncertainty of the average total emissions for 2010–2011 in the southern Amazon and the central South America regions was 7.9 and 13.3 Tg CH_4_ yr^−1^ for APR, 7.7 (-2% for APR) and 13.0 Tg CH_4_ yr^−1^ (-2%) for GB, and 7.0 (-12%) and 11.0 Tg CH_4_ yr^−1^ (-16%) for PST, respectively. These *χ*^2^ statistics and uncertainty reductions indicate that the satellite and ground-based observations constrain the total CH_4_ budget more tightly than the ground-based observations alone, due to their regional and global coverage. The precision improvement of flux estimates provides confidence in our interpretation of observed XCH_4_ variability relative to previous studies that only used ground-based observations. Therefore, the importance of satellite observations for error reduction in inverse modeling is confirmed, especially over regions with fewer ground-based observations, such as the tropics [34].

However, the value of *χ*^2^ = 451 shows that the XCH_4__PST distribution is significantly different to that of the XCH_4_ retrievals (*P* < 0.01), likely due to a combination of factors. The sampling of satellite observations in the southern Amazon region is restricted in the dry season and the spatial distribution differs between 2010 and 2011. These spatio-temporal sampling biases may disturb annual and interannual balances between the dry and wet seasons in the posterior fluxes [35]. The XCH_4_ retrievals also have a higher scatter than ground-based observations because of the inherent limitations of retrieval algorithms [36] and the detection performance of satellite instruments [12], leading to errors in posterior fluxes. Errors also occur in the models, such as uncertainties in a priori fluxes and in the atmospheric transport model. For instance, the a priori total annual fluxes across the southern Amazon and central South America regions for 2010 were higher than those of CarbonTracker-CH_4_ (14.1 and 36.3 Tg CH_4_ yr^−1^, respectively), although posterior fluxes in CarbonTracker-CH_4_ were closer to our results (19.9 and 45.8 Tg CH_4_ yr^−1^). We assigned a relatively small magnitude to the prior flux uncertainty of biomass burning emissions in order to handle prominent fire events in the 2010 dry season. This means that the posterior flux of biomass burning largely relied on the given prior flux. The choice of prior flux uncertainties is critical for posterior fluxes e.g., [37], and previous studies have investigated methods to characterize the uncertainty e.g., [38–40]. However, statistics related to the uncertainty are not fully understood and remain a major challenge in inversion schemes.

To help interpret differences in XCH_4_ variability between 2010 and 2011 and dependence of posterior fluxes on prior ones, we ran the inverse model with identical prior fluxes and uncertainties (Table B in S1 File). Instead of prior fluxes with interannual variations, average seasonal cycle of individual emission sources for the period of 2000–2004 were used as identical prior fluxes for both 2010 and 2011. Uncertainties in the identical prior fluxes were set to 20% of the values for the anthropogenic sources and 50% for the natural and biomass burning sources based on GOSAT Level 4 CH_4_ data product scheme. The results show enhancement of total emissions of 1.4 and 2.6 Tg CH_4_ yr^−1^ in the southern Amazon and central South America regions in 2010 compared with 2011. The enhancement is mainly related to changes in natural emission sources in posterior fluxes due to the largest uncertainty of prior fluxes in three source groups, and there is no clear difference in biomass burning emissions between 2010 and 2011. Although the inverse model with identical prior fluxes perform less well in this test as shown in small uncertainty reductions, the test reveals the limitation of our ability to quantify biomass burning emissions during Amazonian drought in 2010. As these issues associated with errors in posterior fluxes are difficult to take into account and are key limitations in this study, it should be emphasized that the posterior fluxes can vary with the distribution of satellite sampling, the retrieval algorithms, and the models that were used.

To estimate biomass burning emissions with a different way, we analyzed carbon monoxide (CO) concentrations retrieved from the Measurements Of Pollution In The Troposphere (MOPITT) satellite observations [41] (Fig 2c), as CO serves as a proxy for CH_4_ emitted during smoldering [42]. Monthly CO levels increased markedly in the southern Amazon region in 2010, peaking at 290–312 ppb (median monthly values) in August and September. Additionally, seasonal variations in the two regions correspond to the ΔXCH_4_ variations for the two years. These data support the correlation between high-intensity fires and enhanced CH_4_ emissions by biomass burning in the southern Amazon region during the 2010 drought. Smoke from fires in the region was also detectable by visual inspection of coarse-pixel GOSAT cloud imagery data (Fig D in S1 File).

Globally, wetland CH_4_ emissions emerge as the primary driver of variability in atmospheric CH_4_ [43]. In wetlands, the water table depth is a key determinant of emission rates, along with temperature and substrate availability [44]. Increased levels of solar radiation during droughts may partly increase CH_4_ emissions from wetlands, by increasing the amount of substrate available for methanogenesis due to Amazonian forests green-up [45] and increasing temperature in the soil and water. However, during the 2010 drought, low water levels caused by unusually low precipitation resulted in a smaller flooded area [46], thus promoting aerobic soil conditions and reducing CH_4_ production. In our estimate, the reduced CH_4_ emissions from wetlands caused by the drought occurred beyond the dry season in the southern Amazon region (10.2 ± 4.2 and 11.1 ± 3.9 Tg CH_4_ for 2010 and 2011 respectively). Consequently, wetland dynamics is unlikely to explain the enhanced CH_4_ emissions in 2010. Drier soil conditions may stimulate termite activity by presenting greater volumes of decaying root biomass, possibly leading to increased CH_4_ emissions to the atmosphere. However, a current estimate of global emissions from this source is only ∼10 Tg CH_4_ yr^−1^, and the emissions in South America account for about 30% of this total [2, 44]. Thus, this contribution alone cannot explain the increased CH_4_ emissions of ∼4.8 Tg CH_4_ yr^−1^ in the southern Amazon region in 2010.

Anthropogenic emissions are another important source of atmospheric CH_4_. In Brazil, which constitutes the greater part of the southern Amazon region, anthropogenic CH_4_ sources are dominated by emissions from ruminants, which in 2005 accounted for 57% (11.5 Tg CH_4_ yr^−1^) of total CH_4_ emissions [47]. In Brazil, both total and ruminant emissions decreased slightly from 2005 to 2010, by 6% and 13%, respectively, probably because of the global economic recession. Overall, anthropogenic emissions appear to have a minor influence on the CH_4_ budget in the southern Amazon region.

## Summary

Although no definite conclusions about CH_4_ emissions are possible because of the uncertainty in our estimates, our analyses raise the possibility that severe drought events in 2010 influenced CH_4_ emissions in the Amazon. This is apparent as enhanced biomass burning related to land clearing and pasture maintenance [48]. The CH_4_ emitted from biomass burning accumulates as greenhouse gases in the atmosphere, which may further enhance the severity and frequency of drought (although drought can also cause a decline in greenhouse gas emissions from tropical soils, via changes in soil redox potential and nutrient availability [49]). The satellite observations provide a solution for mapping XCH_4_ distributions over the Amazon region, allowing improvements to our knowledge of the carbon budget. Improvements in data acquirement over cloud-covered areas could lead to further constraints of the CH_4_ budget in the region. The overall effects of Amazonian drought on greenhouse gas budgets are not yet fully understood. Further research, such as multi-site investigations of fire regimes and precise ground-based observations of atmospheric CH_4_ emissions, are key to improving our understanding the contribution of Amazonian drought to greenhouse gas emissions.

## Supporting Information

S1 File The supplementary materials for this study. (PDF)
