# Supplementary material for: Enhanced Methane Emissions during Amazonian Drought by Biomass Burning
Source: PLoS One. 2016 Nov 16;11(11):e0166039. doi: 10.1371/journal.pone.0166039 (PMC5113007; doi:10.1371/journal.pone.0166039)
Supplement: S1 File — (PDF) [file pone.0166039.s001.pdf]

Supplementary Materials for
Enhanced methane emissions during Amazonian drought by biomass burning

Makoto Saito,[1*] Heon Sook Kim,[1] Akihiko Ito,[1]
Tatsuya Yokota,[1] Shamil Maksyutov[1]

[1]Center for Global Environmental Research, National Institute for Environmental Studies,
16-2 Onogawa, Tsukuba, Ibaraki 305-8506, Japan

*To whom correspondence should be addressed; E-mail: saito.makoto@nies.go.jp

Table A: Prior and posterior fluxes of total, anthropogenic (ANT), natural (NAT), and biomass burning (BMB) emissions (Tg CH_4 yr^{-1}) and their uncertainties in the southern Amazon and central South America regions.

	Year	Total	ANT	NAT	BMB
Southern Amazon					
prior					
	2010	22.62 ± 8.66	2.90 ± 0.58	13.79 ± 6.89	5.94 ± 1.19
	2011	16.83 ± 7.22	2.90 ± 0.58	12.86 ± 6.43	1.08 ± 0.22
posterior					
	2010	26.60 ± 7.53	3.16 ± 0.53	16.45 ± 5.97	6.99 ± 1.03
	2011	21.83 ± 6.37	3.17 ± 0.53	17.51 ± 5.63	1.14 ± 0.21
Central South America					
prior					
	2010	39.97 ± 13.56	19.99 ± 4.00	18.54 ± 9.27	1.45 ± 0.29
	2011	38.36 ± 13.03	19.99 ± 4.00	17.87 ± 8.93	0.50 ± 0.10
posterior					
	2010	43.93 ± 11.18	20.69 ± 3.84	21.79 ± 7.05	1.45 ± 0.29
	2011	45.04 ± 10.90	20.46 ± 3.87	24.08 ± 6.93	0.51 ± 0.10

Table B: As in Table A but for identical prior fluxes and uncertainties.

	Year	Total	ANT	NAT	BMB
Southern Amazon					
prior					
	2010/2011	17.09 ± 7.68	2.90 ± 0.58	12.85 ± 6.42	1.35 ± 0.67
posterior					
	2010	24.28 ± 7.33	2.95 ± 0.58	19.70 ± 6.10	1.63 ± 0.65
	2011	22.85 ± 7.35	2.94 ± 0.58	18.34 ± 6.11	1.57 ± 0.66
Central South America					
prior					
	2010/2011	39.60 ± 13.80	19.99 ± 4.00	18.93 ± 9.47	0.67 ± 0.34
posterior					
	2010	45.85 ± 11.50	20.89 ± 3.82	24.27 ± 7.35	0.69 ± 0.34
	2011	43.29 ± 11.69	20.20 ± 3.85	22.42 ± 7.50	0.67 ± 0.34

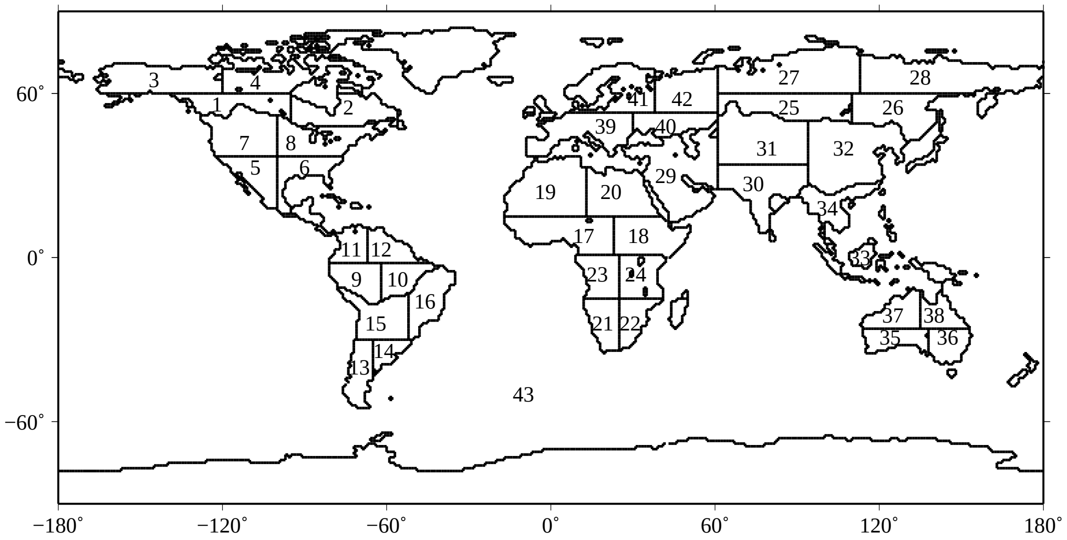

Fig A: Map of the 43 regions analyzed in the study.

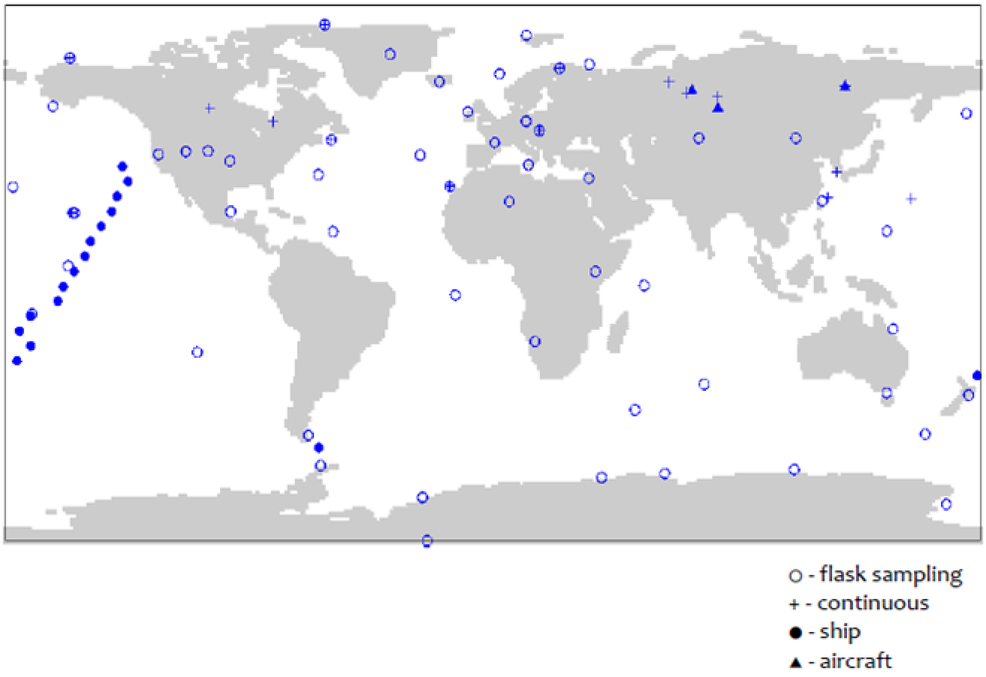

Fig B: Distribution of ground-based observation sites. Open circles show flask sampling sites, crosses show continuous measurement sites, closed circles show ship measurement sites, and closed triangles show aircraft measurement sites.

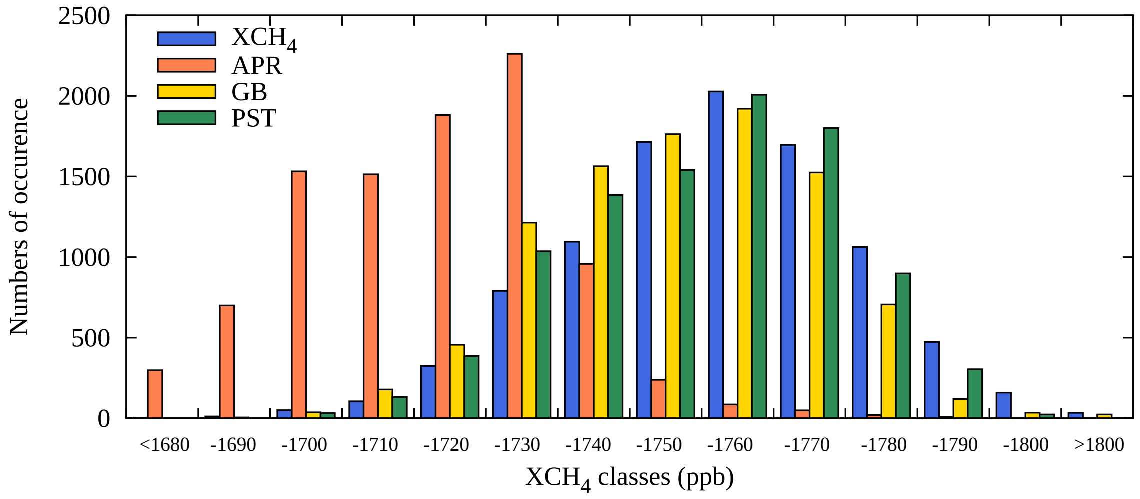

Fig C: Histogram of the 2010–2011 XCH_4 data (ppb) over the southern Amazon and the central South America regions. Blue bars are XCH_4 retrievals, and orange, yellow and green bars are XCH_4 estimated using fluxes for APR, GB and PST, respectively.

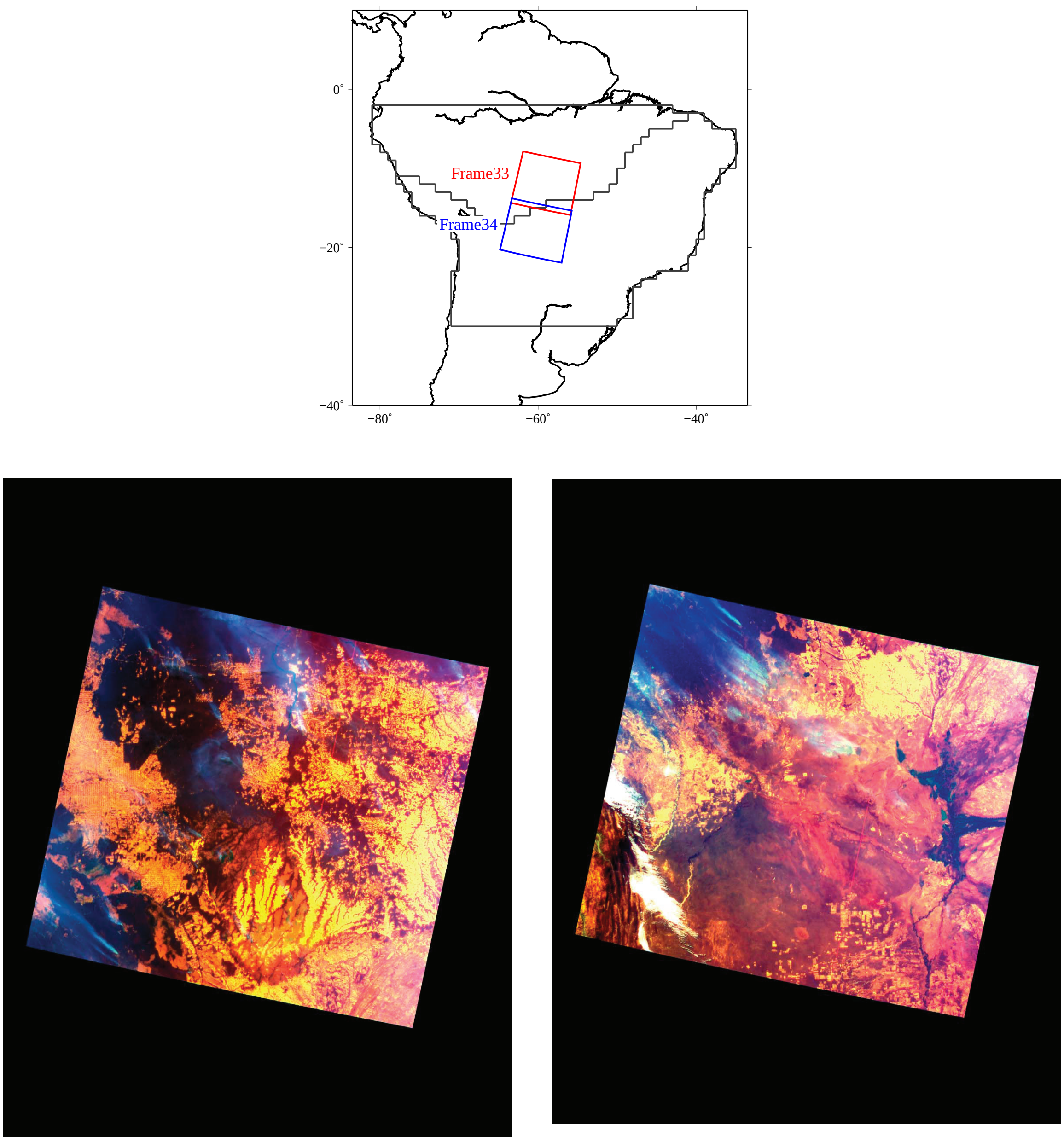

Fig D: Browse images acquired by TANSO-CAI on 15 August 2010 of the southern Amazon (bottom left, Frame33) and central South America (bottom right, Frame34) regions. Color components of the image represent wavelengths of 1.6 μm (red), 0.67 μm (green), and 0.38 μm (blue). Smoke caused by biomass burning is recorded in the northern and southwestern parts of Frame33 and the northwestern part of Frame34.
